# Urine-Based Biomarkers in the Diagnosis of Upper Tract Urothelial Carcinoma: A Systematic Review and Meta-Analysis

**DOI:** 10.3390/jcm15041612

**Published:** 2026-02-19

**Authors:** Hanqing Xiao, Liqi Yi, Zhiyang Ma, Kaihan Dai, Yulai Liu, Yi Gao, Danfeng Xu, Hai Huang

**Affiliations:** 1 Department of Urology, Ruijin Hospital, Shanghai Jiao Tong University School of Medicine, Shanghai 200025, China; 2 Department of Urology, Ruijin Hospital Lu Wan Branch, Shanghai Jiao Tong University School of Medicine, Shanghai 200025, China; 3 Department of Urology, Sanming First Hospital Affiliated to Fujian Medical University, Sanming 365000, China

**Keywords:** UTUC, urine test, biomarkers, diagnostic accuracy

## Abstract

**Background:** Upper tract urothelial carcinoma (UTUC) is a rare malignancy with a poor prognosis. Current diagnostic methods such as ureteroscopic biopsy and imaging techniques are invasive, costly, or have low sensitivity. Urine-based biomarkers represent a promising non-invasive alternative. **Objective:** To systematically review and meta-analyze the diagnostic accuracy of various urine-based biomarkers for UTUC and provide a head-to-head comparison. **Methods:** A comprehensive literature search was conducted in PubMed, Scopus, Embase, Cochrane, and Web of Science from January 2000 to October 2025. Studies meeting PICOS criteria were included. Diagnostic performance metrics including sensitivity (SEN), specificity (SPE), positive likelihood ratio (PLR), negative likelihood ratio (NLR), diagnostic odds ratio (DOR), and area under the curve (AUC) were extracted. Data were synthesized using the MIDAS module in StataMP 18.0. **Results:** Seventeen studies involving 2734 patients were included. The overall pooled sensitivity was 0.86, specificity 0.94, diagnostic odds ratio 93, and AUC 0.94. DNA methylation assays showed balanced performance (SEN = 0.89, SPE = 0.92), gene mutation assays had the highest specificity (SPE = 0.97), RNA assays had the highest sensitivity (SEN = 0.97) but low specificity (SPE = 0.25). Significant heterogeneity was observed, but no notable publication bias was detected. **Conclusions:** Urine-based biomarkers demonstrate high diagnostic performance for UTUC, particularly DNA methylation assays. Future prospective studies are warranted to validate their clinical utility.

## 1. Introduction

Upper tract urothelial carcinoma (UTUC) is a relatively rare malignancy, encompassing renal pelvic and ureteral cancers [1], with an annual incidence of approximately 2 per 100,000 individuals, accounting for 5–10% of all urothelial carcinomas in Western countries. Although its incidence is lower than that of bladder cancer, UTUC is often diagnosed at a more advanced stage and carries a poorer prognosis. The 5-year cancer-specific mortality rates for patients with pT2, pT3, and pT4 disease are 21%, 35%, and 59% [2,3], respectively, posing significant challenges for clinical management.

The current diagnostic gold standard for UTUC is ureteroscopic biopsy. Other diagnostic modalities include imaging techniques such as computed tomography urography (CTU), magnetic resonance urography (MRU), or 18F-fluorodeoxyglucose positron emission tomography/computed tomography (FDG-PET/CT), as well as urinary cytology [1]. However, these methods face substantial limitations: urinary cytology suffers from low sensitivity; CTU is ineffective in detecting flat lesions and often cannot reliably distinguish between benign and malignant conditions; while ureteroscopy (URS), despite its diagnostic potential, is costly, technically demanding, invasive, and carries risks of procedure-related complications and potential tumor seeding [4,5,6]. Consequently, due to the inadequacies of existing methods, there is a pressing need for more effective diagnostic and risk-stratification tools for UTUC.

In recent years, numerous urine- and blood-based biomarkers have been investigated as non-invasive alternatives for UTUC diagnosis. Although some biomarkers, such as *FGFR3* [7] and *NMP22* [8], have shown promising results, their reported diagnostic accuracy varies considerably across studies. The existing literature is characterized by small-scale, single-center studies, lacking a comprehensive synthesis of the evidence. Moreover, to the best of our knowledge, a comprehensive diagnostic meta-analysis that directly compares the performance across different categories of biomarkers for UTUC is currently lacking. Previous reviews have often been narrative or focused on a single type of biomarker [9]. Therefore, we conducted this systematic review and meta-analysis to provide a head-to-head comparison of various biomarker classes, thoroughly evaluate their collective and individual diagnostic performance for UTUC, and identify the most promising avenues for future development. We anticipate that the pooled estimates and heterogeneity exploration from this study will aid clinicians in selecting appropriate biomarkers and inform the design of future biomarker discovery and validation studies for UTUC.

## 2. Materials and Methods

The study protocol has been pre-registered in the International Prospective Register of Systematic Reviews (PROSPERO: double-blind peer review policy). This systematic review adheres to the reporting guidelines outlined in the Preferred Reporting Items for Systematic Reviews and Meta-Analyses extension for Network Meta-Analyses (PRISMA-NMA) [10,11], as well as the Assessing the Methodological Quality of Systematic Reviews (AMSTAR) guidelines [12,13]. The PRISMA and AMSTAR checklists are provided in Supplementary Table S1 and Supplementary Material S1, respectively.

### 2.1. Study Selection

Two authors (X.H. and H.H.) conducted a comprehensive literature search using the PubMed, Scopus, Embase, Cochrane, and Web of Science databases for the period from January 2000 to October 2025, with no language restrictions. The search strategy employed a combination of Medical Subject Headings (MeSH) terms and free-text words. Predefined search terms included “DNA”, “RNA”, “biomarker”, “urine test”, “UTUC”, “upper tract urothelial carcinoma”, “upper tract urinary carcinoma”, “urothelial carcinoma”, “renal pelvis tumor”, and “ureter tumor”. Synonyms were connected using the Boolean operator “OR”, while terms representing different concepts were combined using “AND”. All studies pertaining to the epidemiology and diagnostic assessment of upper tract urothelial carcinoma during this period were included. Additional relevant publications were identified by reviewing reference lists and related citation features.

**Figure 1:**
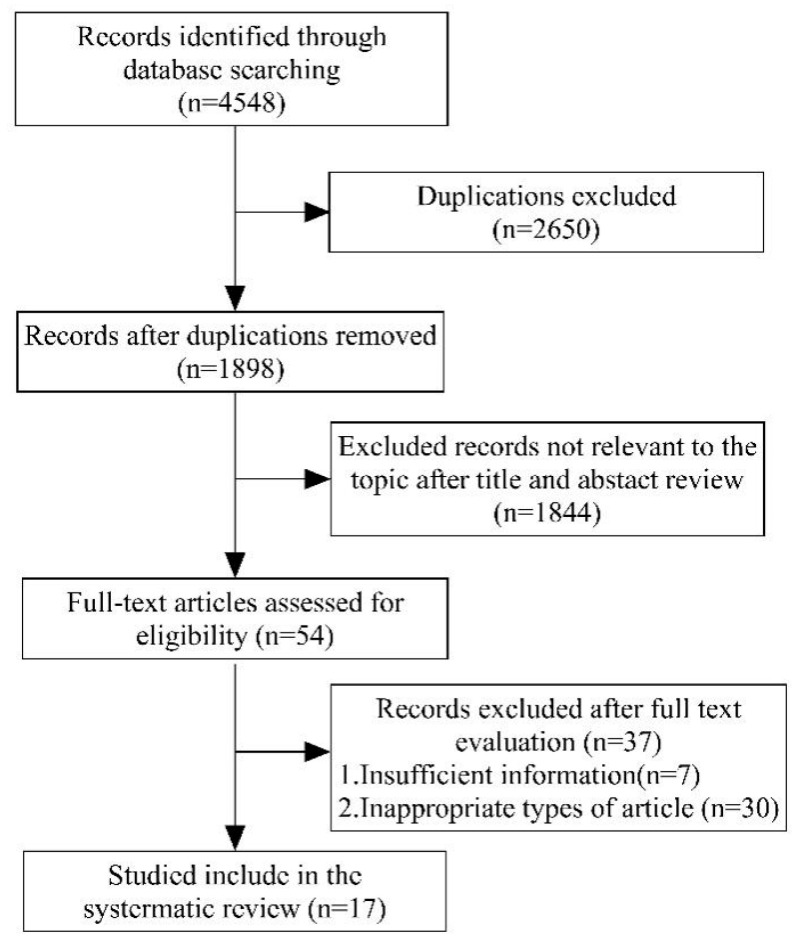
The specific screening PRISMA flow chart of included studies.

Study selection followed the Population, Intervention, Comparator, Outcomes, Study design (PICOS) principles [14]: (1) Population (P): Patients with suspected or confirmed UTUC, (2) Intervention (I): Biomarker-based urine tests, (3) Comparator (C): Pathologically confirmed UTUC group versus non-UTUC control group, (4) Outcomes (O): Sensitivity (SEN), specificity (SPE), positive likelihood ratio (PLR), negative likelihood ratio (NLR), diagnostic odds ratio (DOR), and the area under the summary receiver operating characteristic curve (AUC), (5) Study design (S): No restrictions. Exclusion criteria comprised: (a) studies with insufficient data, (b) unpublished articles, case reports, reviews, letters, single-arm trials, and editorials, and (c) duplicate publications. The study selection process is detailed in Figure 1.

### 2.2. Data Extraction

The following data were independently extracted by two reviewers, with any discrepancies resolved through consensus: (1) first author’s name, (2) year of publication, (3) journal, (4) country where the study was conducted, (5) study period, (6) inclusion criteria, (7) method used for urine test, (8) proportion of high-grade UTUC, (9) urine sample collection method, (10) total number of patients, (11) number of patients with UTUC, (12) number of patients without UTUC (control group), (13) sensitivity, specificity, positive predictive value (PPV), negative predictive value (NPV), and area under the curve (AUC).

### 2.3. Data Consolidation

Data synthesis was performed using the MIDAS module in StataMP software (version 18.0). The diagnostic accuracy was evaluated by pooling the sensitivity (SEN), specificity (SPE), positive likelihood ratio (PLR), negative likelihood ratio (NLR), diagnostic odds ratio (DOR), and their corresponding 95% confidence intervals (CIs). SEN and SPE served as the primary outcome measures in this study. Concurrently, the PLR and NLR were calculated, representing the ratio of the probability of a positive/negative urine test result in the UTUC group to the probability in the non-UTUC group. A pooled PLR > 10 suggests that a positive result is strongly indicative of confirming UTUC, whereas a pooled NLR < 0.1 suggests that a negative result is strongly indicative of ruling out UTUC [15]. Additionally, a summary receiver operating characteristic (SROC) curve was constructed using a bivariate model, and the overall performance of the test was assessed by the area under the curve (AUC). Heterogeneity was evaluated using the Higgins *I*^2^ statistic, with an *I*^2^ value > 50% indicating significant heterogeneity. Potential sources of significant heterogeneity were investigated through subgroup analysis and meta-regression. Study-level covariates were selected based on a full-text review. The covariates in this study included the urine biomarker assay method (categorized as gene mutation assay, DNA methylation assay, RNA assay, or protein/post-translational product assay), patient geographical region (Asia or Western countries), and tumor grade (proportion of high-grade UTUC in the enrolled studies: <60%, ≥60% but ≤80%, or >80%). Publication bias was assessed using Deeks’ funnel plot asymmetry test, with a *p*-value < 0.05 considered indicative of statistically significant publication bias.

The methodological quality of the included studies was assessed using the Quality Assessment of Diagnostic Accuracy Studies (QUADAS-2) tool [16]. If any domain of the QUADAS-2 assessment in a study was identified as having a high risk of bias, the overall risk of bias for that study was judged to be high. Additionally, concerns regarding applicability in the domains of patient selection, index test, and reference standard were evaluated.

## 3. Results

### 3.1. Search Results

The results of the systematic review are presented in Figure 1, and the detailed characteristics are presented in Supplementary Table S2. The initial search using the aforementioned terms across the five databases yielded a total of 4548 records. Following a full-text assessment, only 17 studies met the eligibility criteria for further qualitative analysis. Table 1 summarizes the main characteristics of these 17 studies. Collectively, these studies included 2734 cases, were published between 2001 and 2024, had sample sizes ranging from 41 to 402, and reported patient recruitment periods spanning from 2001 to 2024. Of the included studies, four (4/17, 23.5%) focused on gene mutation assays [7,17,18,19], nine (9/17, 52.9%) investigated DNA methylation assays [17,19,20,21,22,23,24,25,26], three (3/17, 17.6%) examined RNA assays [24,27,28], and four (4/17, 23.5%) explored proteins and post-translational products [29,30,31]. Nearly half of the included studies (7/17, 41.2%) were conducted in China [8,17,19,22,23,27,31]. Three studies investigated multiple categories of urine-based assays [17,19,24]. In total, there were eleven (11/17,64.7%) prospective studies [8,20,21,22,23,24,27,28,29,30,31] and six (6/17, 35.3%) retrospective studies [7,17,18,19,25,26]. Among these, eleven (11/17, 64.7%) were single-center studies [7,17,18,19,20,21,22,23,24,25,26] and six (6/17, 35.3%) were multi-center studies [8,27,28,29,30,31].

### 3.2. Methodological Quality

The overall risk of bias varied across the included studies, with eight studies demonstrating a moderate risk and the remaining nine studies exhibiting a high risk (Supplementary Figure S1). This assessment was conducted using the QUADAS-2 tool, evaluating domains such as patient selection, index test, reference standard, and flow and timing.

### 3.3. Diagnostic Accuracy of Urine Test in UTUC

**Figure 2:**
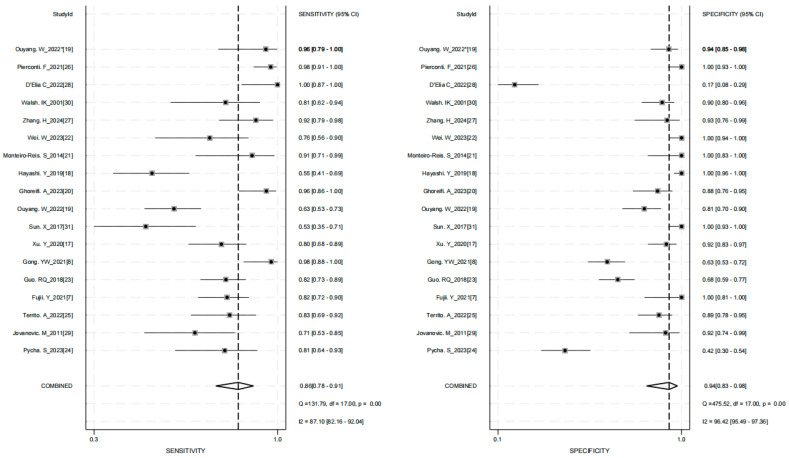
Forest plots about the sensitivity (**Left**) and specificity (**Right**) of urine test in upper tract urothelial carcinoma diagnosis.

**Figure 3:**
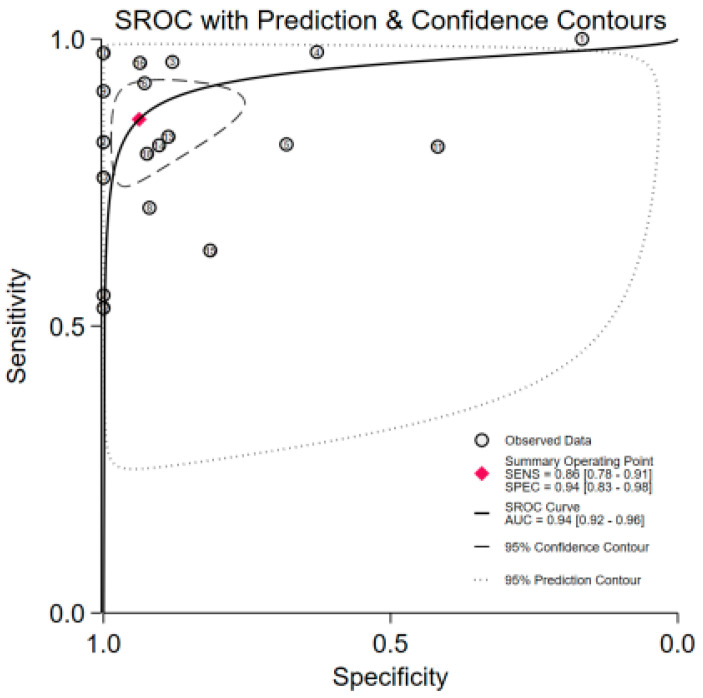
The summary receiver operating characteristic curve of urine test in the diagnosis of upper tract urothelial carcinoma.

The pooled diagnostic performance of various urine assay methods for upper tract urothelial carcinoma (UTUC) was as follows: (1) sensitivity (SEN) = 0.86 (95% CI: 0.78–0.91); specificity (SPE) = 0.94 (95% CI: 0.83–0.98), with significant heterogeneity (*p* < 0.01, *I*^2^ = 87.10% and *p* < 0.01, *I*^2^ = 96.42%) (Figure 2), (2) positive likelihood ratio (PLR) = 13.9 (95% CI: 4.8–40.3); negative likelihood ratio (NLR) = 0.15 (95% CI: 0.09–0.23), with significant heterogeneity (*p* < 0.01, *I*^2^ = 97.58% and *p* < 0.01, *I*^2^ = 87.47%) (Supplementary Figure S2), and (3) diagnostic odds ratio (DOR) = 93 (95% CI: 31–281) (Supplementary Figure S3). (4) AUC = 0.94 (95% CI: 0.92–0.96) (Figure 3). Given that this study involved a substantial number of retrospective and single-center clinical trials, sensitivity analysis addressing this limitation are presented in Table 2. The results demonstrate that the conclusion regarding the significant diagnostic value of urine liquid biopsy for UTUC is robust and reliable.

**Figure 4:**
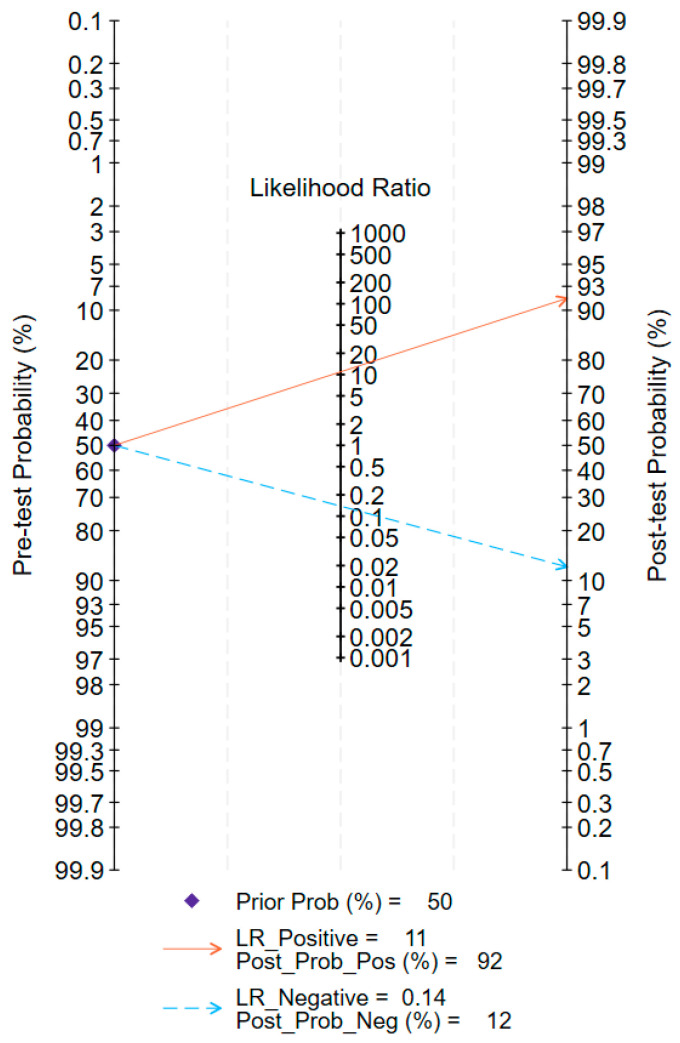
Fagan plot analysis for the evaluation of urine test in upper tract urothelial carcinoma early detection.

**Figure 5:**
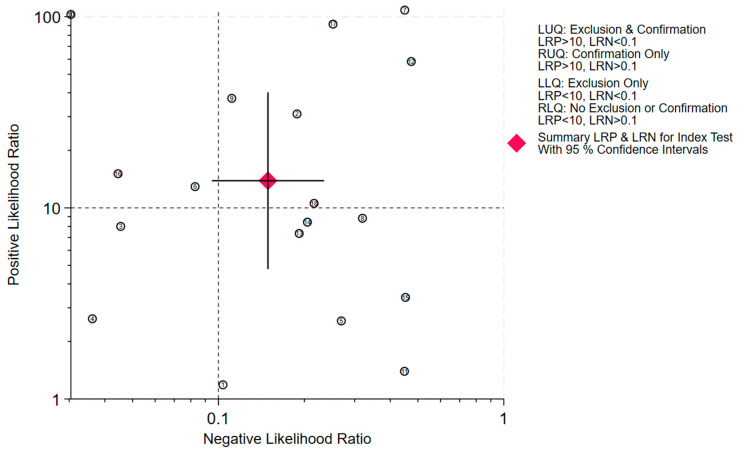
Likelihood ratio scatter plot for the evaluation of urine test in upper tract urothelial carcinoma early detection.

**Figure 6:**
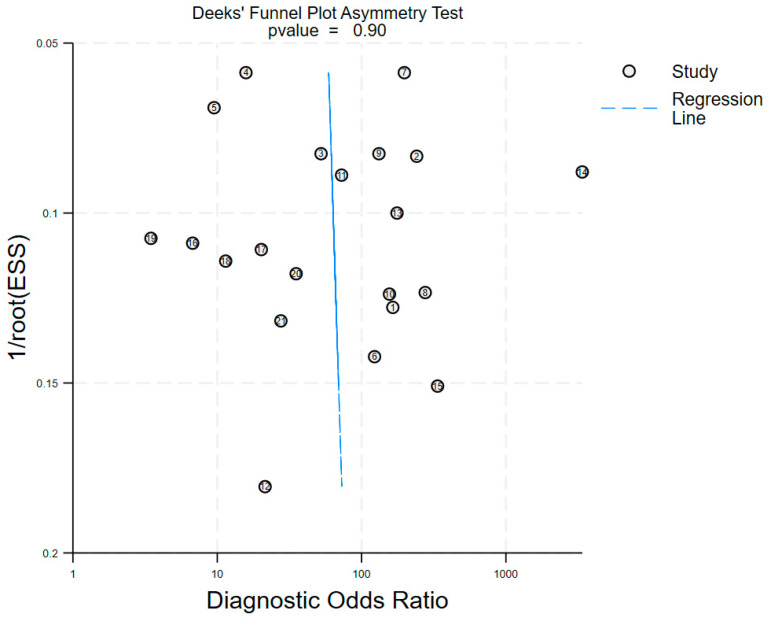
Deeks’ funnel plot for detecting publication bias.

When test results are negative or positive with a pre-test probability of 50%, the post-test probabilities for upper tract urothelial carcinoma (UTUC) are 12% and 92%, respectively (Figure 4). The likelihood ratio scatter plot demonstrated a positive likelihood ratio (PLR) greater than 10 and a negative likelihood ratio (NLR) greater than 0.1 (Figure 5). In Deek’s funnel plot asymmetry test, the *p*-value for the slope coefficient was 0.90, indicating no significant publication bias (Figure 6).

### 3.4. Heterogeneity Investigation

The covariates in the subgroup analysis and meta-regression included liquid assay methods, geographical region, and tumor grade.

Regarding assay methods, 4 studies utilized gene mutation assays, 9 studies employed DNA methylation assays, 3 studies used RNA assays, and 4 studies involved protein and post-translational product assays. The gene mutation assays yielded a pooled sensitivity (SEN) of 0.62 (95% CI: 0.43–0.79) and a pooled specificity (SPE) of 0.97 (95% CI: 0.95–0.99) (Supplementary Figure S4). The DNA methylation assays demonstrated a pooled SEN of 0.89 (95% CI: 0.83–0.93) and a pooled SPE of 0.92 (95% CI: 0.84–0.96) (Supplementary Figure S5). The RNA assays showed a pooled SEN of 96.91% (95% CI: 93.5–100%) and a pooled SPE of 25.32% (95% CI: 18.5–32.2%). The assays for proteins and post-translational products resulted in a pooled SEN of 77.37% (95% CI: 70.4–84.4%) and a pooled SPE of 75.12% (95% CI: 69.1–81.1%). Significant heterogeneity was observed in all these pooled results (*p* < 0.01, *I*^2^ > 50%).

Regarding regional distribution, twelve studies were conducted in Asia, yielding a pooled sensitivity (SEN) of 0.81 (95% CI: 0.69–0.90) and a pooled specificity (SPE) of 0.95 (95% CI: 0.88–0.98) (Supplementary Figure S6). Among the nine studies conducted in Western countries, the pooled SEN was 0.93 (95% CI: 0.80–0.98) and the pooled SPE was 0.83 (95% CI: 0.46–0.96) (Supplementary Figure S7). In the aforementioned analysis, significant heterogeneity was observed (*p* < 0.01, *I*^2^ > 50%).

Furthermore, tumor grade is also worthy of investigation. For clarity of presentation, the proportion of high-grade tumors is defined as follows: low proportion (<60%), intermediate proportion (≥60% but ≤80%), and high proportion (>80%). Six studies were categorized into the low proportion group, demonstrated a pooled sensitivity of 0.91 (95% CI: 0.67–0.98) and a pooled specificity of 0.63 (95% CI: 0.22–0.91) (Supplementary Figure S8). Seven studies were categorized into the intermediate proportion group, demonstrated a pooled sensitivity of 0.80 (95% CI: 0.60–0.92) and a pooled specificity of 0.92 (95% CI: 0.81–0.97) (Supplementary Figure S9). Seven studies were categorized into the high proportion group, demonstrated a pooled sensitivity of 0.89 (95% CI: 0.76–0.95) and a pooled specificity of 0.98 (95% CI: 0.88–1.00) (Supplementary Figure S10). Significant heterogeneity was observed among the analysis (*p* < 0.01, *I*^2^ > 50%).

Moreover, we performed meta-regression analyses on these covariates to identify potential sources of heterogeneity. However, the analyses yielded no significant results, indicating that the substantial heterogeneity observed could not be explained by the current subgroup and meta-regression analyses (Supplementary Figure S11).

## 4. Discussion

Upper tract urothelial carcinoma (UTUC) poses a significant diagnostic challenge in urology due to its deep anatomical location, difficulties in early detection, and poor prognosis. The current diagnostic gold standard, ureteroscopic biopsy, while accurate, is invasive and carries risks of bleeding, perforation, and even tumor seeding. Imaging techniques and urinary cytology are limited by insensitivity for flat lesions and low overall sensitivity, respectively. Consequently, there is an urgent clinical need for the development of highly accurate, non-invasive diagnostic tools for UTUC. This systematic review and meta-analysis provide, for the first time, a comprehensive head-to-head comparison of the performance of various urine-based biomarker categories for UTUC diagnosis, aiming to offer high-level evidence for clinical practice and future research.

Our meta-analysis demonstrates that urine-based biomarkers exhibit outstanding overall diagnostic performance for UTUC, with a pooled sensitivity (SEN) of 0.86, specificity (SPE) of 0.94, a diagnostic odds ratio (DOR) as high as 93, and an area under the summary receiver operating characteristic curve (AUC) of 0.94. These results are statistically highly significant (all 95% confidence intervals are far from the null value), indicating the substantial clinical potential of urine tests as a non-invasive diagnostic tool for UTUC. To contextualize the added value of these emerging urine-based biomarkers, it is informative to compare their performance with that of conventional urinary cytology, which remains a widely used non-invasive adjunct in clinical practice. Traditional voided urinary cytology, while offering high specificity (typically >90%), suffers from notoriously low and variable sensitivity for UTUC, with reported values ranging from 30% to 50%, and being particularly poor for low-grade tumors [6,32,33]. In stark contrast, the pooled sensitivity of the urine biomarkers analyzed in this study is 0.86, representing a substantial (approximately 2–3 fold) improvement in detection capability. Although the pooled specificity (0.94) is comparable to that of cytology, the key advancement lies in achieving this high specificity while dramatically increasing sensitivity. This translates to a significantly higher negative predictive value, meaning a negative biomarker test is far more reliable than a negative cytology result for ruling out disease. Specifically, the pooled positive likelihood ratio (PLR) of 13.9 means that a positive urine test result is nearly 14 times more likely in a patient with UTUC than in a non-UTUC individual; according to the Fagan nomogram, with a pre-test probability of 50%, a positive test increases the probability of cancer to 92%. However, the pooled negative likelihood ratio (NLR) of 0.15, while indicating that a negative result significantly reduces the probability of disease (from 50% to 12%), does not reach the “ideal” threshold of <0.1. This implies that a single urine biomarker test, in its current stage, cannot completely replace invasive procedures for “ruling out” diagnosis. Its value in avoiding unnecessary invasive procedures lies more in initial screening and auxiliary decision-making for high-risk populations.

A deeper analysis of different biomarker categories reveals performance variations with clear clinical implications:

DNA methylation assays were the most numerous among the included studies (9 studies) and showed excellent diagnostic performance (SEN = 0.89, SPE = 0.92). The high sensitivity suggests its effectiveness in detecting the majority of UTUC patients, including some with early-stage or low-grade tumors, while the high specificity helps avoid false positives, reducing unnecessary follow-up and invasive procedures for non-cancerous individuals. This balanced performance makes it the most promising single category for current clinical application. For instance, the study by Ouyang et al. [19] combined mutation and methylation, while the prospective study by Ghoreifi et al. [20] validated the reliability of specific methylation markers, laying the groundwork for clinical translation.

Gene Mutation Assays (e.g., *FGFR3*, *TERT*) demonstrated unparalleled specificity (SPE = 0.97). This means that the detection of a specific mutation is strongly indicative of UTUC, providing extremely high value in confirmatory diagnosis, especially when imaging findings are atypical. However, its relatively low sensitivity (SEN = 0.62) is a major limitation, implying that nearly 40% of patients could be missed. Therefore, mutation testing is more suitable as a confirmatory tool or in combination with other highly sensitive markers, rather than as a standalone initial screening tool. Studies by Fujii [7] and Hayashi [18] highlighted the driver role and diagnostic specificity of *FGFR3* and *TERT* mutations in UTUC.

RNA assays presented a distinctive “high-sensitivity, low-specificity” profile (SEN = 0.97, SPE = 0.25). While the near-perfect sensitivity suggests that a negative RNA test can be powerful in ruling out UTUC, the extremely low specificity of approximately 25% constitutes a major clinical limitation. This means that in a population with a 50% pre-test probability, around 75% of positive test results would be false positives.

The clinical implications of this high false-positive rate are substantial and cannot be overlooked: (1) Patient Harm: Patients receiving a false-positive result may experience significant and prolonged psychological distress, anxiety, and the stigma of a potential cancer diagnosis. (2) Unnecessary Medical Interventions: A positive RNA test, given its poor specificity, is insufficient to confirm UTUC. However, in clinical practice, it is likely to trigger a cascade of further investigations, including repeated biomarker tests, advanced imaging (CTU/MRU), and ultimately, diagnostic ureteroscopy—an invasive procedure with inherent risks of bleeding, infection, perforation, and tumor seeding. Thus, a standalone positive RNA test may lead to unnecessary invasive procedures in a large proportion of patients without the disease. (3) Economic Burden: This cascade of low-yield investigations generates substantial and avoidable healthcare costs.

Therefore, in its current form, the clinical utility of RNA assays as a standalone diagnostic tool is severely limited. Crucially, a positive RNA test result should never be the sole basis for proceeding to invasive intervention. Future development must focus on improving the specificity of RNA-based panels, possibly through the discovery of more specific RNA markers or by combining RNA signatures with other biomarker classes in integrated algorithms. Protein and Post-Translational Product Assays showed moderate performance (SEN = 0.77, SPE = 0.75). While their diagnostic accuracy is superior to traditional cytology, they no longer hold a clear advantage over nucleic acid-based markers. Their strengths lie in mature detection technology, low cost, and rapid turnaround time, making them potentially useful in resource-limited settings.

Significant heterogeneity (*I*^2^ > 87%) was observed in this study, which is common in diagnostic meta-analyses. We explored this in depth through pre-specified subgroup analyses and meta-regression. Geographical analysis revealed that studies from Western countries had higher sensitivity (0.93 vs. 0.81), while Asian studies had better specificity (0.95 vs. 0.83). This may reflect differences in genetic backgrounds, environmental exposures, tumor biology, and healthcare referral patterns between Eastern and Western populations. Tumor grade analysis uncovered an important trend: as the proportion of high-grade tumors in the studies increased (from <60% to >80%), the diagnostic specificity significantly improved (from 0.63 to 0.98). This aligns with clinical expectations, as high-grade tumors typically shed more biomarkers and are morphologically more distinct from benign conditions, making them easier to identify accurately. Unfortunately, the meta-regression did not pinpoint a single source of heterogeneity, suggesting it likely stems from a combination of factors.

First, the composition of control populations varied considerably among studies, ranging from healthy volunteers to patients with benign urological conditions (e.g., stones, inflammation) or other malignancies. This spectrum directly impacts the specificity estimates, as distinguishing UTUC from other active urological diseases is inherently more challenging. Second, substantial differences existed in urine collection, processing, and storage protocols, including variables such as urine volume, timing of collection, centrifugation methods, and the targeted fraction (cellular pellet vs. cell-free DNA/RNA). Such procedural inconsistencies can profoundly affect biomarker stability and detection. Third, the analytical platforms and diagnostic thresholds (cut-off values) were highly heterogeneous, utilizing diverse technologies (e.g., different PCR assays, sequencing platforms) with study-specific criteria for a “positive” result. Finally, other unmeasured clinical factors, such as patient recruitment settings and disease stage distribution, may have further contributed.

Additionally, this study has several other limitations. Firstly, the number of included studies and sample sizes, particularly for categories like RNA and mutation assays, could be larger. Secondly, the included literature included over one-third of retrospective studies and approximately two-thirds of single-center studies, which constitutes an important methodological limitation. Most of the primary studies relied on data collected from a single institution, which may not be representative of the broader UTUC patient population due to local referral patterns, diagnostic protocols, and demographic characteristics. This limits the external validity (generalizability) of their findings. More importantly, the retrospective design is susceptible to various biases, most notably selection bias.

However, sensitivity analyses confirmed the robustness of our findings. Excluding all retrospective studies yielded results consistent with the primary analysis, indicating that the core conclusion is primarily driven by prospective evidence and thus holds greater clinical generalizability. Furthermore, diagnostic performance showed no significant difference between single- and multi-center studies, and the pooled effect estimates remained stable upon exclusion of either group. This suggests consistent performance of liquid biopsy across healthcare settings of varying scales. Although study design contributed to heterogeneity, our integrated analysis supports the reliability of the core conclusion that urinary liquid biopsy has significant diagnostic value for UTUC.

Although our analysis did not show significant publication bias statistically, the inherent limitations of the primary study designs suggest that the pooled estimates presented here should be interpreted with caution. They likely represent a ‘best-case’ scenario of biomarker performance that requires validation in prospective, multicenter, and consecutively enrolled cohorts.

**Figure 7:**
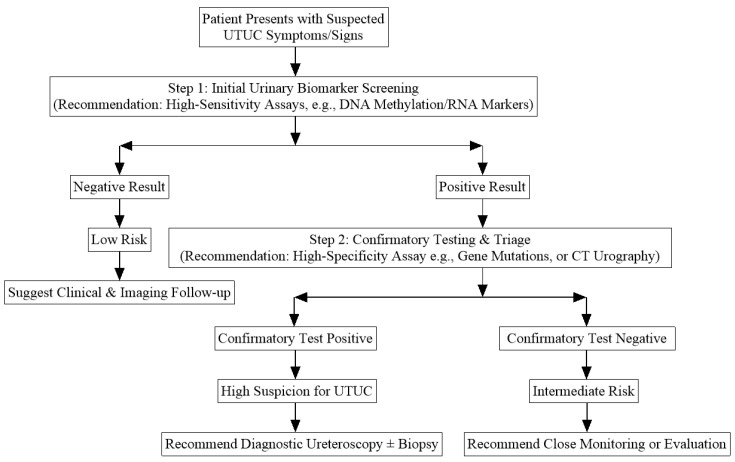
Proposed diagnostic algorithm for suspected Upper Tract Urothelial Carcinoma (UTUC) integrating urine-based biomarkers.

Based on the performance profiles elucidated in this meta-analysis, we propose a sequential diagnostic algorithm to optimize the use of urine biomarkers in the clinical evaluation of suspected UTUC (Figure 7). The goal is to leverage the strengths of different biomarker classes while mitigating their weaknesses, thereby maximizing diagnostic efficiency and minimizing unnecessary invasive procedures.

Step 1 (Initial Triage): For a patient with clinical suspicion of UTUC (e.g., unexplained hematuria), a high-sensitivity urine test—such as a multitarget DNA methylation assay (pooled SEN 0.89, SPE 0.92)—should be considered as the first-line non-invasive investigation. A negative result from this step, given the high sensitivity, can effectively rule out a significant proportion of patients, assigning them to a low-risk category suitable for regular surveillance, thus potentially avoiding immediate invasive workup.

Step 2 (Confirmation/Stratification): A positive result from the initial test should not be equated with a diagnosis of UTUC, given the potential for false positives, especially if using an RNA-based test. These patients should proceed to a confirmatory stage employing a method with high specificity. Options include a second, highly specific biomarker test (e.g., a gene mutation assay like *FGFR3/TERT*, SPE 0.97) or direct anatomical imaging via CT urography (CTU). This step aims to “filter out” false positives from the initial screen.

Step 3 (Definitive Diagnosis): Patients who test positive in this confirmatory stage have a high probability of UTUC and should be referred for diagnostic ureteroscopy with biopsy, the current gold standard for definitive histological diagnosis and treatment planning. Patients with negative confirmatory tests despite a positive initial screen represent an intermediate-risk group requiring closer clinical and imaging follow-up.

This proposed pathway emphasizes that urine biomarkers are best positioned as adjuncts within a sequential diagnostic framework, not as standalone replacements for current standards. Clinical judgment, patient preferences, and resource availability must guide the final application of any such algorithm. Prospective validation of this or similar integrated approaches is a crucial next step for translational research.

Looking ahead, the application prospects for urine biomarkers in UTUC management are vast. Firstly, there is a pressing need for large-scale, multicenter, prospective cohort studies to validate the clinical utility of existing promising markers (especially DNA methylation panels). Secondly, exploring optimal combinations of different biomarker categories holds promise for developing diagnostic algorithms with both high sensitivity and specificity. Furthermore, the value of these biomarkers in post-operative surveillance, treatment response evaluation, and prognosis prediction warrants further investigation.

## 5. Conclusions

This systematic review and meta-analysis confirms that urine-based biomarkers exhibit excellent overall diagnostic performance for UTUC as a non-invasive tool. Among the different biomarker categories, DNA methylation assays demonstrate the most favorable balance between sensitivity and specificity, highlighting their significant potential for clinical translation. Gene mutation tests possess exceptionally high specificity, making them ideal for confirmatory diagnosis, while RNA assays, with their superior sensitivity, hold considerable value for ruling out disease, albeit currently limited by their lower specificity.

Significant heterogeneity was identified and explored, potentially originating from geographical variations, tumor grade, and assay methodologies, underscoring the need for standardized protocols and reporting in future research. Despite these variations, urine-based biomarkers, particularly nucleic acid-based tests, offer a promising avenue to overcome the limitations of traditional diagnostic methods for UTUC. Future efforts should focus on validating these biomarkers through large-scale prospective trials and exploring their integration into staged diagnostic algorithms, such as the one proposed herein, to ultimately achieve precise, risk-adapted, and less invasive management for UTUC.

## Figures and Tables

**Table 1:** The characteristics of the included studies.

Study & Year & Country	Test Method	Participants & Performance Metrics
Ghoreifi. A & 2023 & USA [20]	DNA methylation assay	Total (Cases/Controls): 100 (50/50) High-Grade Tumor Proportion: 0.82 Performance (SEN/SPE): 0.96/0.88
Pierconti. F & 2021 & Italy [26]	DNA methylation assay	Total (Cases/Controls): 134 (82/52) High-Grade Tumor Proportion: 1 Performance (SEN/SPE): 0.97/1
Guo. RQ & 2018 & China [23]	DNA methylation assay	Total (Cases/Controls): 211 (98/113) High-Grade Tumor Proportion: 0.631 Performance (SEN/SPE): 0.82/0.68
Monteiro-Reis. S & 2013 & Portugal [21]	DNA methylation assay	Total (Cases/Controls): 42 (22/20) High-Grade Tumor Proportion: 0.86 Performance (SEN/SPE): 0.91/1
Pycha. S & 2023 & Switzerland [24]	DNA methylation assay	Total (Cases/Controls): 97 (31/66) High-Grade Tumor Proportion: 0.161 Performance (SEN/SPE): 0.65/0.79
Territo. A & 2022 & Spain [25]	DNA methylation assay	Total (Cases/Controls): 83 (47/36) High-Grade Tumor Proportion: 0.426 Performance (SEN/SPE): 0.83/0.81
Ouyang. W & 2022 & China [19]	DNA methylation assay	Total (Cases/Controls): 402 (95/307) High-Grade Tumor Proportion: 0.8 Performance (SEN/SPE): 0.92/0.95
Ouyang. W * & 2022 & China [19]	DNA methylation assay	Total (Cases/Controls): 76 (24/52) High-Grade Tumor Proportion: NA Performance (SEN/SPE): 0.96/0.92
Wei. W & 2023 & China [22]	DNA methylation assay	Total (Cases/Controls): 49 (29/20) High-Grade Tumor Proportion: 0.897 Performance (SEN/SPE): 0.76/1
Xu. Y & 2020 & China [17]	DNA methylation assay	Total (Cases/Controls): 150 (64/86) High-Grade Tumor Proportion: 0.734 Performance (SEN/SPE): 0.89/0.94
D’Elia. C & 2022 & Italy [28]	RNA assay	Total (Cases/Controls): 87 (27/60) High-Grade Tumor Proportion: 0.26 Performance (SEN/SPE): 1/0.17
Pycha. S & 2023 & Switzerland [24]	RNA assay	Total (Cases/Controls): 97 (31/66) High-Grade Tumor Proportion: 0.161 Performance (SEN/SPE): 1/0.05
Zhang. H & 2024 & China [27]	RNA assay	Total (Cases/Controls): 67 (39/28) High-Grade Tumor Proportion: 0.8974 Performance (SEN/SPE): 0.92/0.93
Fujii. Y & 2021 & Japan [7]	Gene mutation assay	Total (Cases/Controls): 96 (78/18) High-Grade Tumor Proportion: 0.548 Performance (SEN/SPE): 0.82/1
Hayashi. Y & 2019 & Japan [18]	Gene mutation assay	Total (Cases/Controls): 153 (56/97) High-Grade Tumor Proportion: 0.839 Performance (SEN/SPE): 0.55/1
Xu. Y & 2020 & China [17]	Gene mutation assay	Total (Cases/Controls): 150 (64/86) High-Grade Tumor Proportion: 0.734 Performance (SEN/SPE): 0.72/0.95
Ouyang. W & 2022 & China [19]	Gene mutation assay	Total (Cases/Controls): 402 (95/307) High-Grade Tumor Proportion: 0.8 Performance (SEN/SPE): 0.35/0.97
Walsh. IK & 2001 & UK [30]	BTA stat test	Total (Cases/Controls): 81 (27/54) High-Grade Tumor Proportion: 0.926 Performance (SEN/SPE): 0.82/0.89
Gong. YW & 2021 & China [8]	BTA stat test	Total (Cases/Controls): 157 (44/113) High-Grade Tumor Proportion: 0.659 Performance (SEN/SPE): 0.98/0.63
Jovanovic. M & 2011 & Serbia [29]	NMP22	Total (Cases/Controls): 59 (34/25) High-Grade Tumor Proportion: 0.3529 Performance (SEN/SPE): 0.71/0.92
Sun. X & 2017 & China [31]	p16/Ki-67	Total (Cases/Controls): 41 (32/9) High-Grade Tumor Proportion: 0.75 Performance (SEN/SPE): 0.53/1

The data of this line (marked *) was from an external evaluation group of Wei’s study published in 2022.

**Table 2:** The sensitivity analysis for included studies.

Without Study	Sensitivity (95% CI)	Specificity (95% CI)	Diagnostic Odds Ratio (95% CI)
None	86.0% (78.0–91.0)	94.0% (83.3–97.9)	93.2 (31.3–281.2)
Retrospective Studies	84.7% (79.3–89.0)	76.8% (66.5–84.7)	18.2 (9.9–33.4)
Prospective Studies	74.8% (61.9–84.6)	96.3% (87.7–99.0)	67.5 (20.3–224.5)
Multi-Center Studies	83.7% (76.8–89.0)	83.2% (72.2–90.5)	24.6 (11.8–51.4)
Single-Center Studies	76.0% (65.1–84.6)	87.9% (74.6–94.7)	23.3 (8.0–67.8)

## Data Availability

No new data were created or analyzed in this study. Data sharing is not applicable to this article.

## Supplementary Materials

The following supporting information can be downloaded at: https://www.mdpi.com/article/10.3390/jcm15041612/s1, Supplementary Table S1. The PRISMA checklist of the present study; Supplementary Table S2. The detailed characteristics of the included studies; Supplementary Figure S1. The Quality Assessment of Diagnostic Accuracy Studies for assessing the quality of included studies; Supplementary Figure S2. Forest plots about the PLR (Left) and NLR (Right) of urine test in upper tract urothelial carcinoma diagnosis; Supplementary Figure S3. Forest plots about the diagnostic score (Left) and diagnostic advantage ratio (Right) of urine test in upper tract urothelial carcinoma diagnosis; Supplementary Figure S4. Forest plots about the SEN (Left) and SPE (Right) of gene mutation test in upper tract urothelial carcinoma diagnosis; Supplementary Figure S5. Forest plots about the SEN (Left) and SPE (Right) of DNA methylation test in upper tract urothelial carcinoma diagnosis; Supplementary Figure S6. Forest plots about the SEN (Left) and SPE (Right) of urine test in upper tract urothelial carcinoma diagnosis. (Asian); Supplementary Figure S7. Forest plots about the SEN (Left) and SPE (Right) of urine test in upper tract urothelial carcinoma diagnosis. (Western); Supplementary Figure S8. Forest plots about the SEN (Left) and SPE (Right) of urine test in upper tract urothelial carcinoma diagnosis. (low proportion group); Supplementary Figure S9. Forest plots about the SEN (Left) and SPE (Right) of urine test in upper tract urothelial carcinoma diagnosis. (intermediate proportion group); Supplementary Figure S10. Forest plots about the SEN (Left) and SPE (Right) of urine test in upper tract urothelial carcinoma diagnosis. (high proportion group); Supplementary Figure S11. The results of meta-regression analysis about the investigation of the potential source of heterogeneity. Supplementary Material S1. The AMSTAR checklist of the present study.
