# Supplementary material for: Urine-Based Biomarkers in the Diagnosis of Upper Tract Urothelial Carcinoma: A Systematic Review and Meta-Analysis
Source: J Clin Med. 2026 Feb 19;15(4):1612. doi: 10.3390/jcm15041612 (PMC12941476; doi:10.3390/jcm15041612)
Supplement: Supplementary file 1 [file jcm-15-01612-s001.zip › jcm-4099614-supplementary.pdf]

Supplementary list

Supplementary Table S1. The PRISMA checklist of the present study.

Section and Topic	Item #	Checklist item	Location where item is reported
TITLE			
Title	1	Identify the report as a systematic review.	Page 1
ABSTRACT			
Abstract	2	See the PRISMA 2020 for Abstracts checklist.	Page 1
INTRODUCTION			
Rationale	3	Describe the rationale for the review in the context of existing knowledge.	Page 1
Objectives	4	Provide an explicit statement of the objective(s) or question(s) the review addresses.	Page 1
METHODS			
Eligibility criteria	5	Specify the inclusion and exclusion criteria for the review and how studies were grouped for the syntheses.	Page 2
Information sources	6	Specify all databases, registers, websites, organisations, reference lists and other sources searched or consulted to identify studies. Specify the date when each source was last searched or consulted.	Page 2
Search strategy	7	Present the full search strategies for all databases, registers and websites, including any filters and limits used.	Page 2
Selection process	8	Specify the methods used to decide whether a study met the inclusion criteria of the review, including how many reviewers screened each record and each report retrieved, whether they worked independently, and if applicable, details of automation tools used in the process.	Page 2
Data collection process	9	Specify the methods used to collect data from reports, including how many reviewers collected data from each report, whether they worked independently, any processes for obtaining or confirming data from study investigators, and if applicable, details of automation tools used in the process.	Page 2

Section and Topic	Item #	Checklist item	Location where item is reported
Data items	10a	List and define all outcomes for which data were sought. Specify whether all results that were compatible with each outcome domain in each study were sought (e.g. for all measures, time points, analyses), and if not, the methods used to decide which results to collect.	Page 2
	10b	List and define all other variables for which data were sought (e.g. participant and intervention characteristics, funding sources). Describe any assumptions made about any missing or unclear information.	Page 2
Study risk of bias assessment	11	Specify the methods used to assess risk of bias in the included studies, including details of the tool(s) used, how many reviewers assessed each study and whether they worked independently, and if applicable, details of automation tools used in the process.	Page 2
Effect measures	12	Specify for each outcome the effect measure(s) (e.g. risk ratio, mean difference) used in the synthesis or presentation of results.	Page 2
Synthesis methods	13a	Describe the processes used to decide which studies were eligible for each synthesis (e.g. tabulating the study intervention characteristics and comparing against the planned groups for each synthesis (item #5)).	Page 2
	13b	Describe any methods required to prepare the data for presentation or synthesis, such as handling of missing summary statistics, or data conversions.	Page 2
	13c	Describe any methods used to tabulate or visually display results of individual studies and syntheses.	Page 2
	13d	Describe any methods used to synthesize results and provide a rationale for the choice(s). If meta-analysis was performed, describe the model(s), method(s) to identify the presence and extent of statistical heterogeneity, and software package(s) used.	Page 2
	13e	Describe any methods used to explore possible causes of heterogeneity among study results (e.g. subgroup analysis, meta-regression).	Page 2
	13f	Describe any sensitivity analyses conducted to assess robustness of the synthesized results.	Page 2
Reporting bias assessment	14	Describe any methods used to assess risk of bias due to missing results in a synthesis (arising from reporting biases).	Page 2
Certainty assessment	15	Describe any methods used to assess certainty (or confidence) in the body of evidence for an outcome.	Page 2

Section and Topic	Item #	Checklist item	Location where item is reported
RESULTS			
Study selection	16a	Describe the results of the search and selection process, from the number of records identified in the search to the number of studies included in the review, ideally using a flow diagram.	Page 3
	16b	Cite studies that might appear to meet the inclusion criteria, but which were excluded, and explain why they were excluded.	Page 3
Study characteristics	17	Cite each included study and present its characteristics.	Page 3
Risk of bias in studies	18	Present assessments of risk of bias for each included study.	Page 3
Results of individual studies	19	For all outcomes, present, for each study: (a) summary statistics for each group (where appropriate) and (b) an effect estimate and its precision (e.g. confidence/credible interval), ideally using structured tables or plots.	Page 3
Results of syntheses	20a	For each synthesis, briefly summarise the characteristics and risk of bias among contributing studies.	Page 3
	20b	Present results of all statistical syntheses conducted. If meta-analysis was done, present for each the summary estimate and its precision (e.g. confidence/credible interval) and measures of statistical heterogeneity. If comparing groups, describe the direction of the effect.	Page 3
	20c	Present results of all investigations of possible causes of heterogeneity among study results.	Page 3
	20d	Present results of all sensitivity analyses conducted to assess the robustness of the synthesized results.	Page 3
Reporting biases	21	Present assessments of risk of bias due to missing results (arising from reporting biases) for each synthesis assessed.	Page 3
Certainty of evidence	22	Present assessments of certainty (or confidence) in the body of evidence for each outcome assessed.	Page 3
DISCUSSION			

Section and Topic	Item #	Checklist item	Location where item is reported
Discussion	23a	Provide a general interpretation of the results in the context of other evidence.	Page 4
	23b	Discuss any limitations of the evidence included in the review.	Page 4
	23c	Discuss any limitations of the review processes used.	Page 4
	23d	Discuss implications of the results for practice, policy, and future research.	Page 4
OTHER INFORMATION			
Registration and protocol	24a	Provide registration information for the review, including register name and registration number, or state that the review was not registered.	Page 2
	24b	Indicate where the review protocol can be accessed, or state that a protocol was not prepared.	Page 2
	24c	Describe and explain any amendments to information provided at registration or in the protocol.	Page 5
Support	25	Describe sources of financial or non-financial support for the review, and the role of the funders or sponsors in the review.	Page 5
Competing interests	26	Declare any competing interests of review authors.	Page 5
Availability of data, code and other materials	27	Report which of the following are publicly available and where they can be found: template data collection forms; data extracted from included studies; data used for all analyses; analytic code; any other materials used in the review.	Page 5

From: Page MJ, McKenzie JE, Bossuyt PM, Boutron I, Hoffmann TC, Mulrow CD, et al. The PRISMA 2020 statement: an updated guideline for reporting systematic reviews. BMJ 2021;372:n71. doi: 10.1136/bmj.n71. This work is licensed under CC BY 4.0. To view a copy of this license, visit https://creativecommons.org/licenses/by/4.0/

Supplementary Table S2. The detailed characteristics of the included studies.

Study	Year	Journal	Country	Period	Inclusion cateria	Total patients	UTUC	Non-UTUC	High-grade	Test method	SEN	SPE	PPV	NPV	AUC	Sample
Ghoreifi. A[20]	2023	The Journal of Urology	USA	2019-2022	Confirmed UTUC	100	50	50	82%	DNA methylation （Bladder CARE Test）	0.96	0.88	0.89	0.96	0.97	Nonselective
Pierconti. F[26]	2021	Human Pathology	Italy	2018-2020	Confirmed UTUC with high-grade	134	82	52	100%	DNA methylation （Bladder Epicheck test）	0.97	1.00	1.00	0.96	NA	Selective
Guo. RQ[23]	2018	Urologic Oncology	China	2016-2017	Confirmed UTUC	211	98	113	63.10%	DNA methylation （CDH1、VIM、RASSF1A、HSPA2、GDF15、TMEFF2）	0.82	0.68	0.69	0.81	0.84	Nonselective;50ml
Monteiro-Reis. S[21]	2013	European Journal of Cancer	Portugal	2006-2012	Confirmed UTUC	42	22	20	86%	DNA methylation （GDF15、TMEFF2 and VIM）	0.91	1.00	1.00	0.91	0.92	Nonselective
Pycha. S[24]	2023	World Journal of Urology	Switzerland	NA	Suspicion of UTUC	97	31	66	16.10%	DNA methylation （Bladder Epicheck test)	0.65	0.79	0.59	0.83	NA	Selective
Territo. A[25]	2022	The journal of urology	Spain	2019-2020	Suspicion of UTUC	83	47	36	42.60%	DNA methylation （Bladder Epicheck test）	0.83	0.81	0.85	0.78	NA	Selective;>10ml
Ouyang. W[19]	2022	Cancers	China	NA	Gross or microscopic hematuria	402	95	307	80%	DNA methylation （NRN1）	0.92	0.95	0.85	0.97	0.93	Nonelective;50ml
Ouyang. W[19]*	2022		China			76	24	52	NA	DNA methylation （NRN1）	0.96	0.92	0.85	0.98	0.97	
Wei. W[22]	2023	American Journal of Clinical Pathology	China	2019-2022	Gross or microscopic hematuria	49	29	20	89.70%	DNA methylation （ONECUT2）	0.76	1.00	1.00	0.74	0.88	Nonselective;80ml

Xu. Y[17]	2020	Frontiers in Oncology	China	2017-2018	Gross or microscopic hematuria	150	64	86	73.40%	DNA methylation （ONECUT2）	0.89	0.94	0.92	0.92	0.93	Nonselective;50ml
D'Elia C[28]	2022	Ther Adv Uro	Italy	NA	Suspicion of UTUC	87	27	60	26%	RNA （Xpert Bladder Cancer Detection）	1.00	0.17	0.35	1.00	0.63	Nonselective；4-5ml
Pycha. S[24]	2023	World Journal of Urology	Switzerland	NA	Suspicion of UTUC	97	31	66	16.10%	RNA （Xpert Bladder Cancer Detection）	1.00	0.05	0.33	1.00	NA	Selective
Zhang. H [27]	2024	Eur Urol Oncol	China	2022-2023	Confirmed UTUC	67	39	28	89.74%	RNA （CA9、CCL18、ERBB2、IGF2、MMP12、PPP1R14D、SGK2、SWINGN）	0.92	0.93	0.95	0.93	0.93	Nonelective;60-80ml
Fujii. Y[7]	2021	Cancer Cell	Japan	NA	Confirmed UTUC	96	78	18	54.80%	Gene mutation （ TP53, FGFR3, HRAS,etc.）	0.82	1.00	1.00	0.65	NA	Nonselective;20ml
Hayashi. Y[18]	2019	Cancer Science	Japan	2013–2019	Confirmed UTUC	153	56	97	83.90%	Gene mutation (TERT+FGFR3)	0.55	1.00	1.00	0.67	NA	Nonselective；4-32ml
Xu. Y[17]	2020	Frontiers in Oncology	China	2017-2018	Gross or microscopic hematuria	150	64	86	73.40%	Gene mutation （AKT1,ASXL2,CREBBP,etc.)	0.72	0.95	0.92	0.82	NA	Nonselective;50ml
Ouyang. W[19]	2022	Cancers	China	NA	Gross or microscopic hematuria	402	95	307	80%	Gene mutation (TERT+FGFR3)	0.35	0.97	0.77	0.83	0.66	Nonelective;50ml

Walsh. IK[30]	2001	Urology	UK	NA	Confirmed UTUC	81	27	54	92.60%	BTA stat test （CFHrp）	0.82	0.89	0.79	0.91	NA	Nonselective
Gong.YW[8]	2021	Translational Cancer Research	China	2015–2017	Confirmed UTUC	157	44	113	65.90%	BTA-stat (CFHrp)	0.98	0.63	0.69	0.97	0.81	Nonselective;10ml
Jovanovic. M[29]	2011	Urologia Internationalis	Serbia	2005-2009	Confirmed UTUC	59	34	25	35.29%	NMP22	0.71	0.92	0.92	0.70	NA	Nonselective
Sun. X[31]	2017	Diagnostic Cytopathology	China	2010-2016	Confirmed UTUC	41	32	9	75%	p16/Ki-67	0.53	1.00	1.00	0.38	NA	Nonelective;50ml

AUC, area under the curve; NA, not available; NLR, negative predictive value; PPV, positive predictive value; SEN, sensitivity; SPE, specificity; UTUC, upper tract urothelial carcinoma.

The data of this line (marked *) was from an external evaluation group of Wei’s study published in 2022.

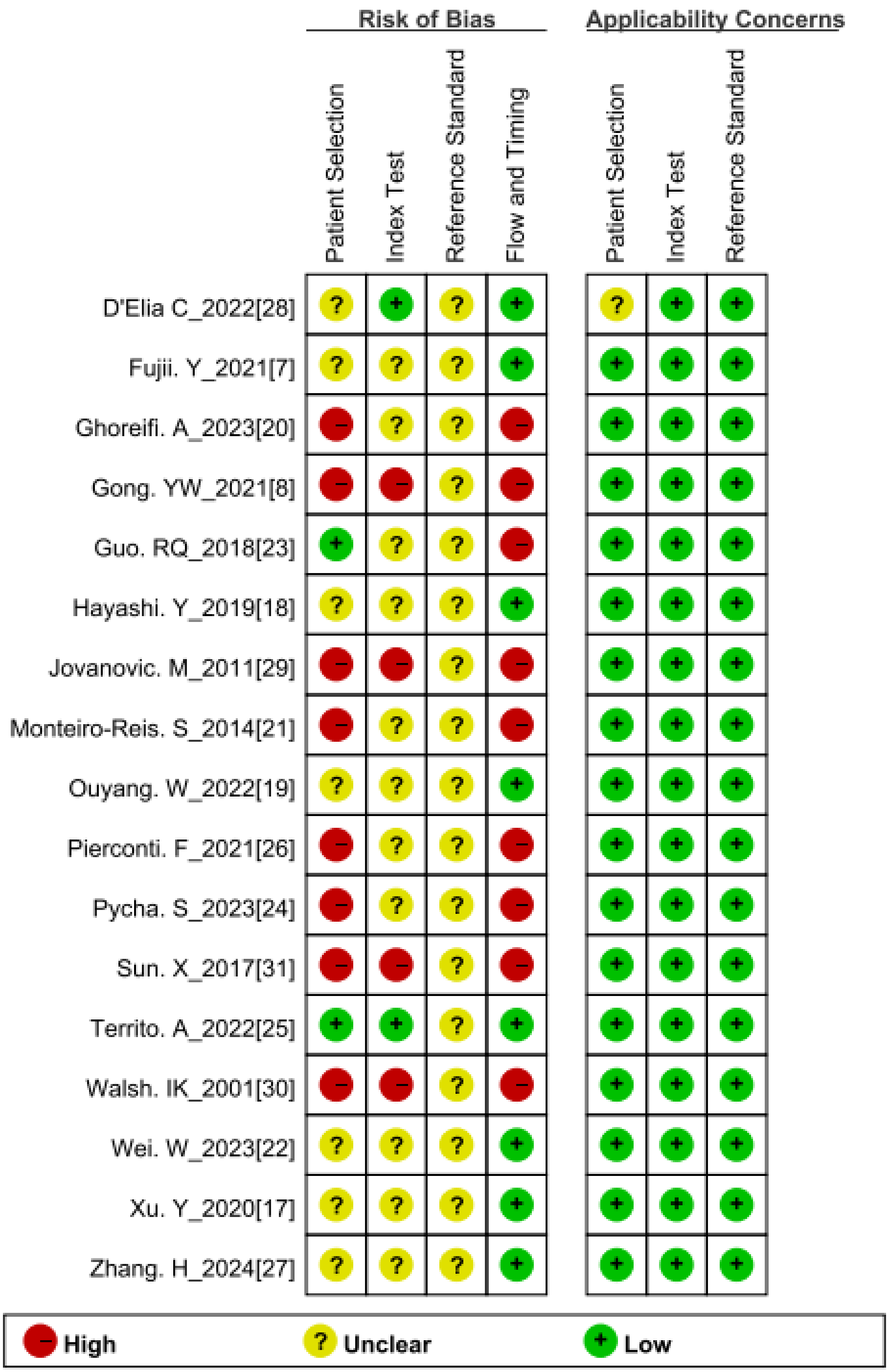

Supplementary Figure S1. The Quality Assessment of Diagnostic Accuracy Studies for assessing the quality of included studies.

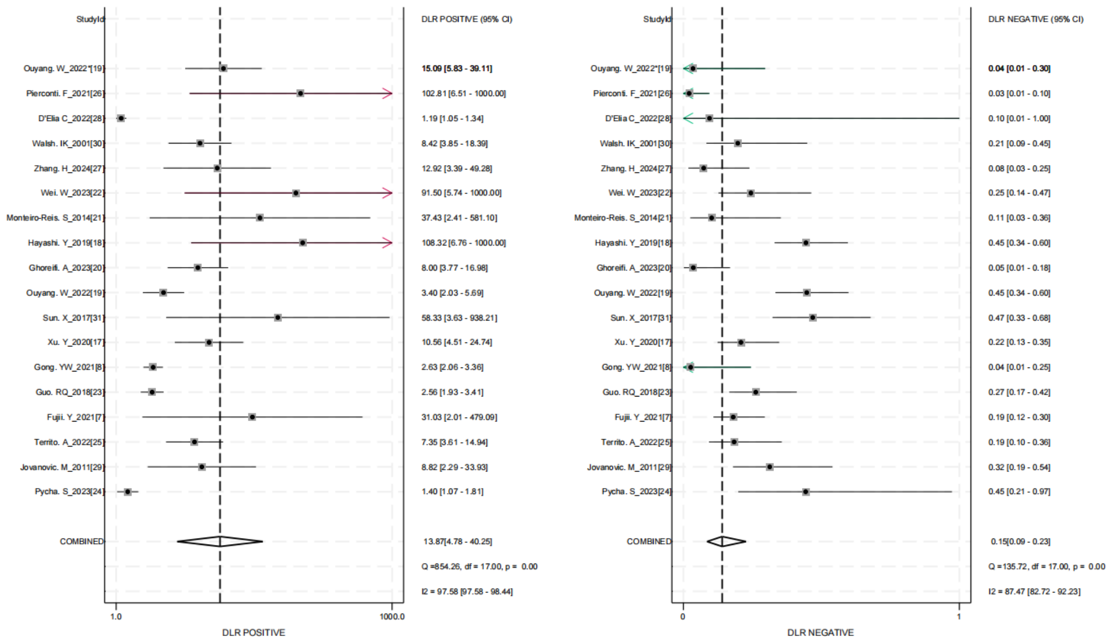

Supplementary Figure S2. Forest plots about the PLR (Left) and NLR (Right) of urine test in upper tract urothelial carcinoma diagnosis.

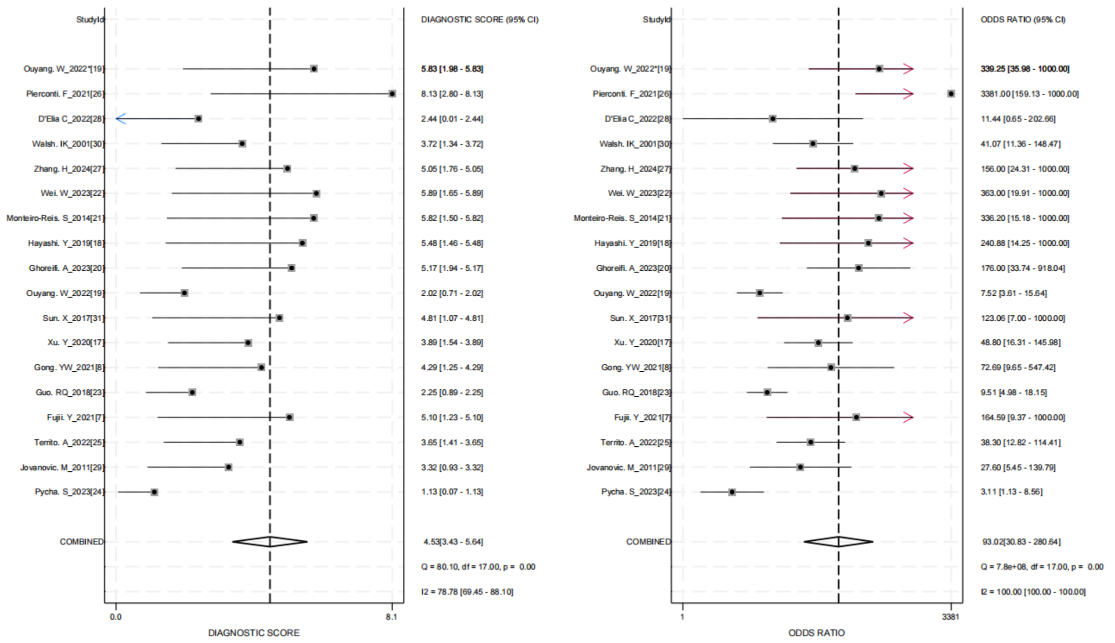

Supplementary Figure S3. Forest plots about the diagnostic score (Left) and diagnostic advantage ratio (Right) of urine test in upper tract urothelial carcinoma diagnosis.

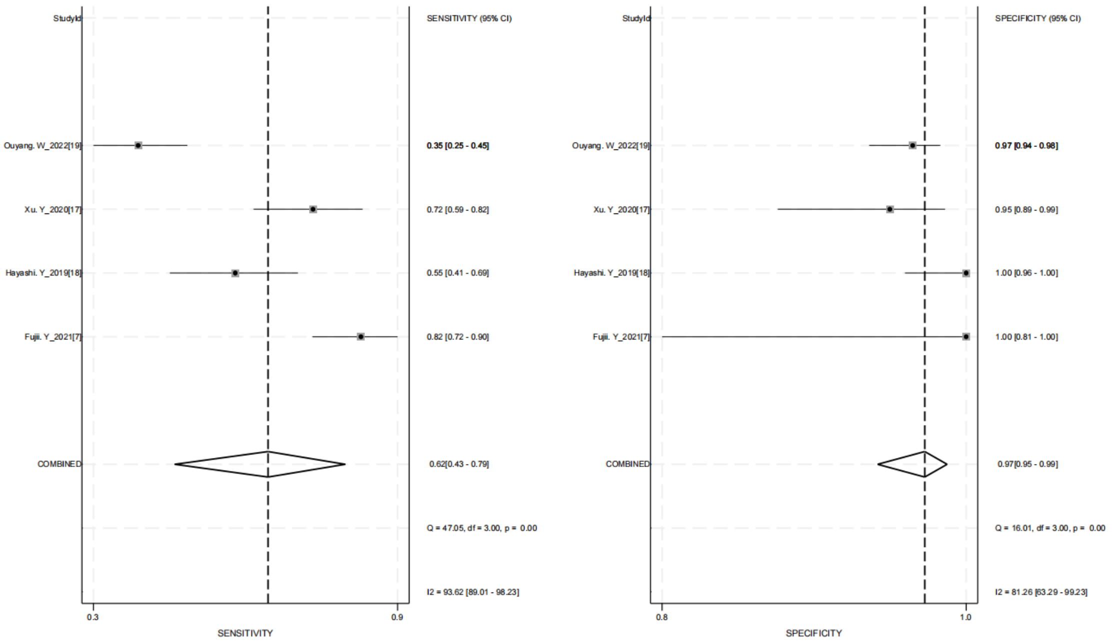

Supplementary Figure S4. Forest plots about the SEN (Left) and SPE (Right) of gene mutation test in upper tract urothelial carcinoma diagnosis.

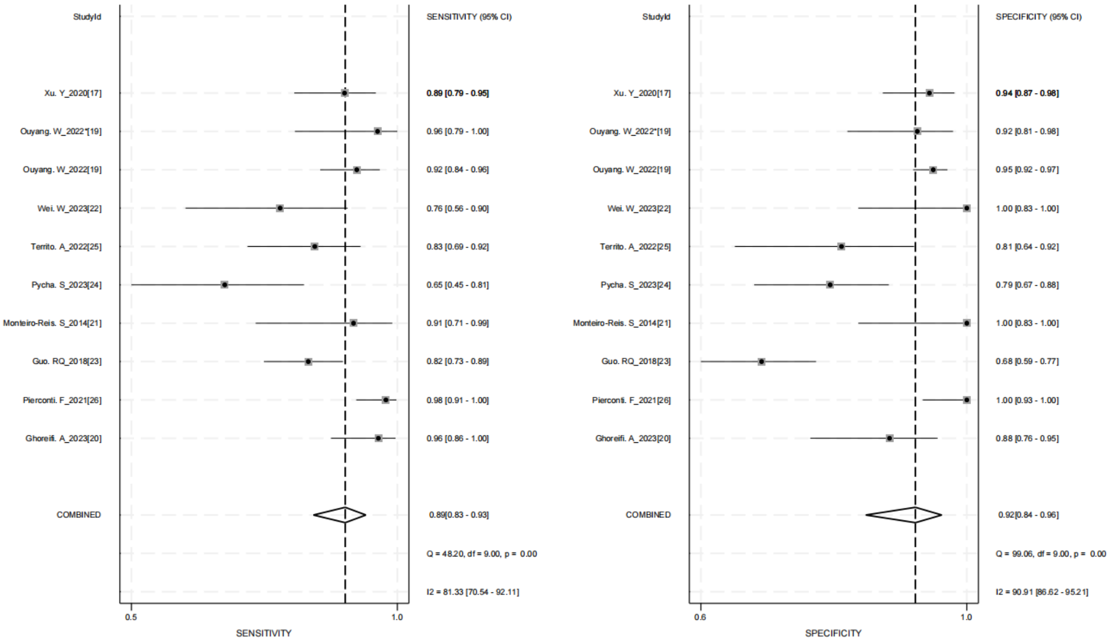

Supplementary Figure S5. Forest plots about the SEN (Left) and SPE (Right) of DNA methylation test in upper tract urothelial carcinoma diagnosis.

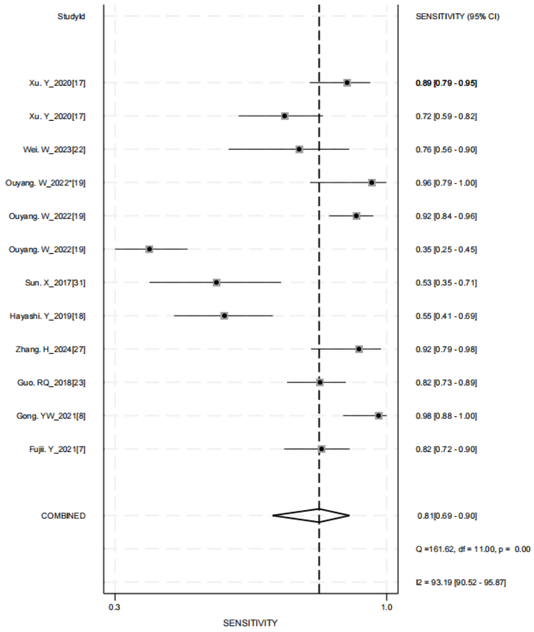

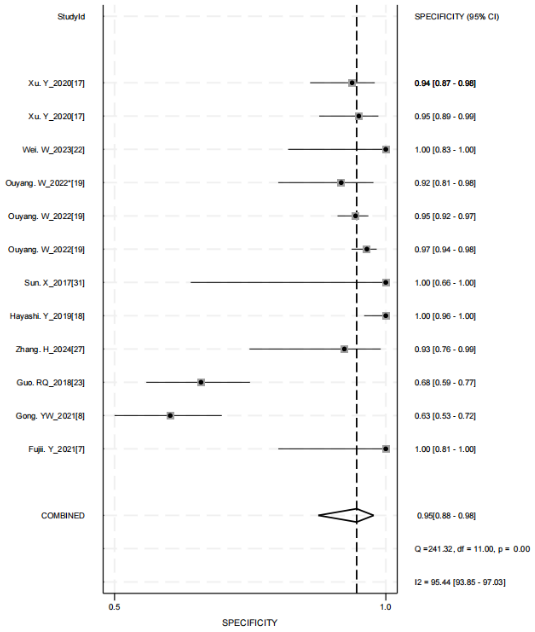

Supplementary Figure S6. Forest plots about the SEN (Left) and SPE (Right) of urine test in upper tract urothelial carcinoma diagnosis. (Asian)

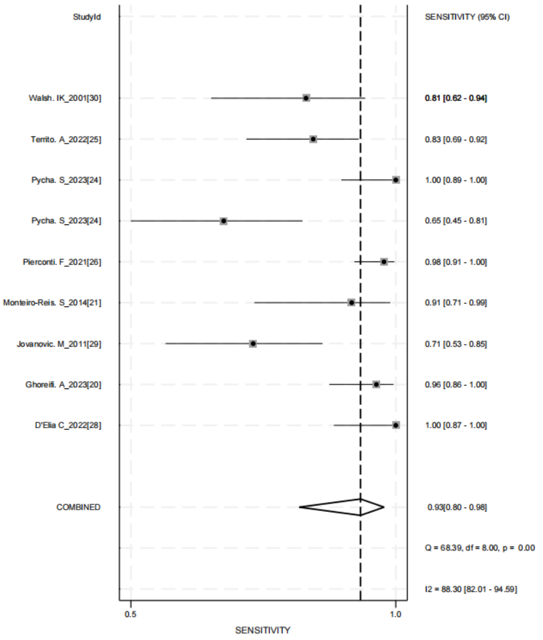

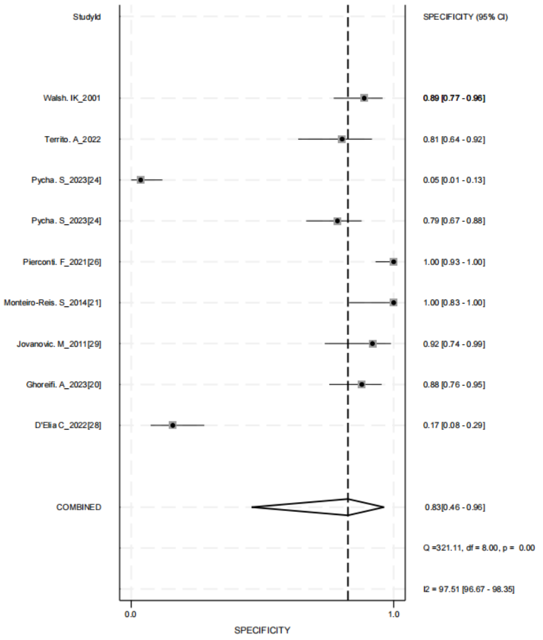

Supplementary Figure S7. Forest plots about the SEN (Left) and SPE (Right) of urine test in upper tract urothelial carcinoma diagnosis. (Western)

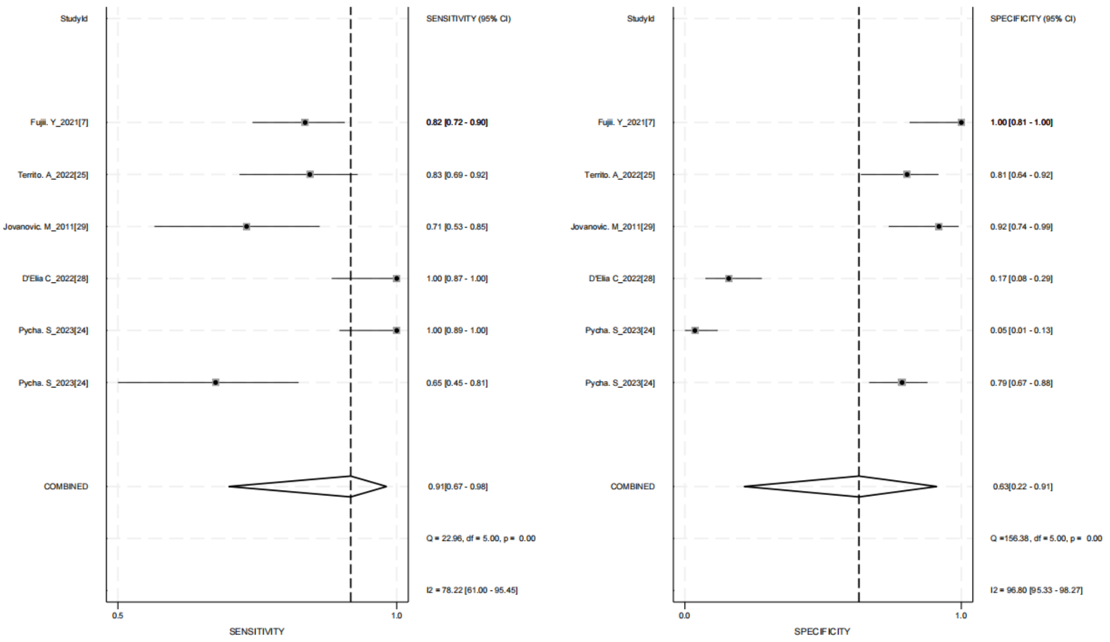

Supplementary Figure S8. Forest plots about the SEN (Left) and SPE (Right) of urine test in upper tract urothelial carcinoma diagnosis. (low proportion group)

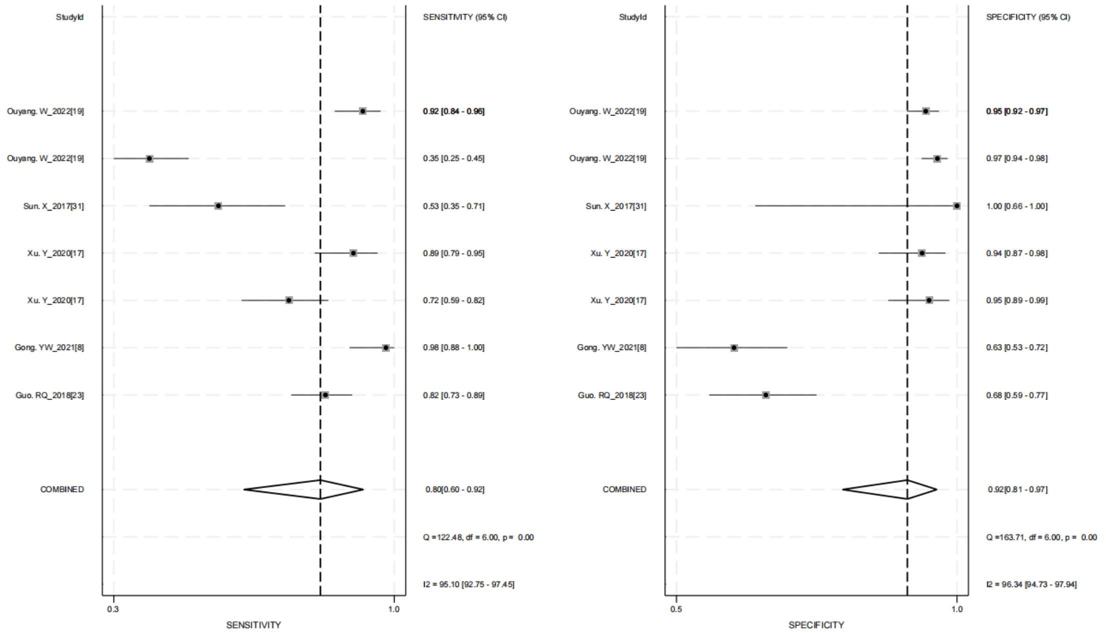

Supplementary Figure S9. Forest plots about the SEN (Left) and SPE (Right) of urine test in upper tract urothelial carcinoma diagnosis. (intermediate proportion group)

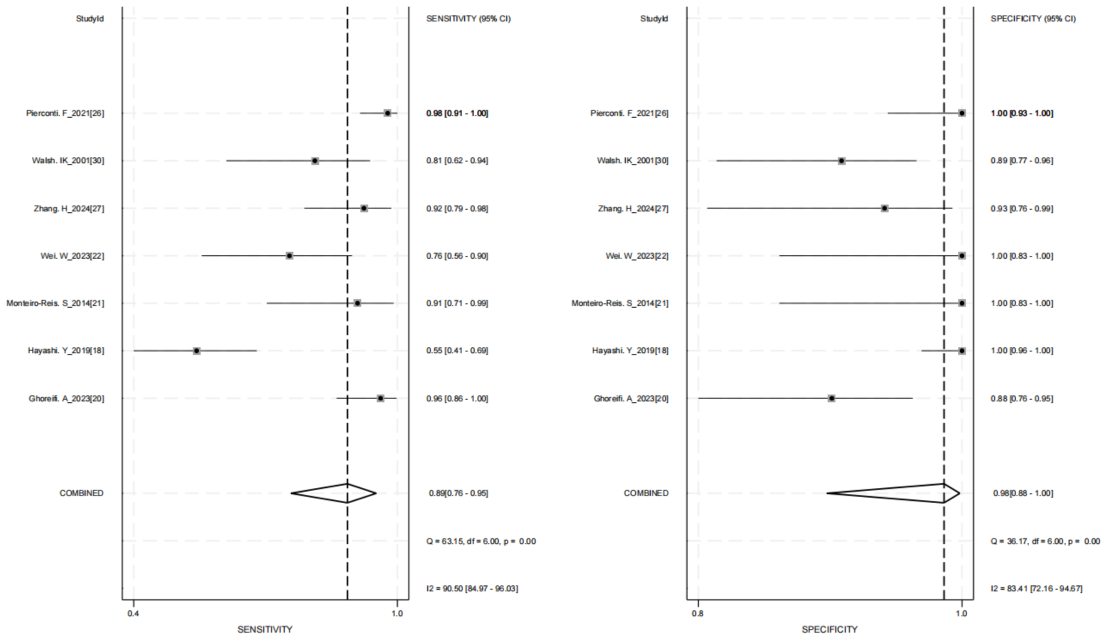

Supplementary Figure S10. Forest plots about the SEN (Left) and SPE (Right) of urine test in upper tract urothelial carcinoma diagnosis. (high proportion group)

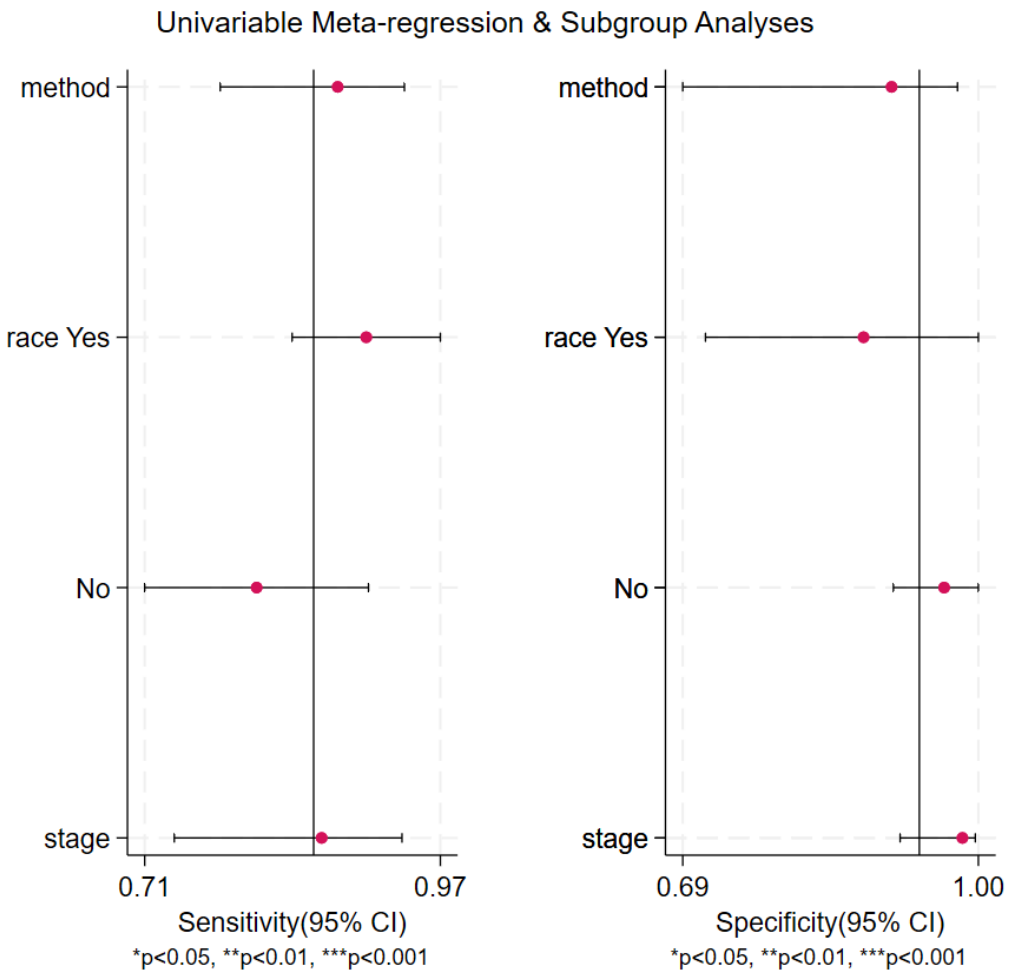

Supplementary Figure S11. The results of meta-regression analysis about the investigation of the potential source of heterogeneity.

Supplementary Material S1. The AMSTAR checklist of the present study.

AMSTAR 2: a critical appraisal tool for systematic reviews that include randomised or non-randomised studies of healthcare interventions, or both

1. Did the research questions and inclusion criteria for the review include the components of PICO?

For Yes:
- ☑ Population
- ☑ Intervention
- ☑ Comparator group
- ☑ Outcome

Optional (recommended)
- ☐ Timeframe for follow-up

- ☑ Yes
- ☐ No

2. Did the report of the review contain an explicit statement that the review methods were established prior to the conduct of the review and did the report justify any significant deviations from the protocol?

For Partial Yes:
The authors state that they had a written protocol or guide that included ALL the following:
- ☐ review question(s)
- ☐ a search strategy
- ☐ inclusion/exclusion criteria
- ☐ a risk of bias assessment

For Yes:
As for partial yes, plus the protocol should be registered and should also have specified:
- ☑ a meta-analysis/synthesis plan, if appropriate, *and*
- ☑ a plan for investigating causes of heterogeneity
- ☑ justification for any deviations from the protocol

- ☑ Yes
- ☐ Partial Yes
- ☐ No

3. Did the review authors explain their selection of the study designs for inclusion in the review?

For Yes, the review should satisfy ONE of the following:
- ☐ *Explanation for* including only RCTs
- ☐ OR *Explanation for* including only NRSI
- ☑ OR *Explanation for* including both RCTs and NRSI

- ☑ Yes
- ☐ No

4. Did the review authors use a comprehensive literature search strategy?

For Partial Yes (all the following):
- ☐ searched at least 2 databases (relevant to research question)
- ☐ provided key word and/or search strategy
- ☐ justified publication restrictions (e.g. language)

For Yes, should also have (all the following):
- ☑ searched the reference lists / bibliographies of included studies
- ☑ searched trial/study registries
- ☑ included/consulted content experts in the field
- ☑ where relevant, searched for grey literature
- ☑ conducted search within 24 months of completion of the review

- ☑ Yes
- ☐ Partial Yes
- ☐ No

5. Did the review authors perform study selection in duplicate?

For Yes, either ONE of the following:
- ☑ at least two reviewers independently agreed on selection of eligible studies and achieved consensus on which studies to include
- ☐ OR two reviewers selected a sample of eligible studies and achieved good agreement (at least 80 percent), with the remainder selected by one reviewer.

- ☑ Yes
- ☐ No

AMSTAR 2: a critical appraisal tool for systematic reviews that include randomised or non-randomised studies of healthcare interventions, or both

11. If meta-analysis was performed did the review authors use appropriate methods for statistical combination of results?

RCTs

For Yes:

- ☐ The authors justified combining the data in a meta-analysis
  - ☐ AND they used an appropriate weighted technique to combine study results and adjusted for heterogeneity if present.
  - ☐ AND investigated the causes of any heterogeneity

- ☐ Yes
- ☐ No
- ☒ No meta-analysis conducted

For NRSI

For Yes:

- ☒ The authors justified combining the data in a meta-analysis
  - ☒ AND they used an appropriate weighted technique to combine study results, adjusting for heterogeneity if present
  - ☒ AND they statistically combined effect estimates from NRSI that were adjusted for confounding, rather than combining raw data, or justified combining raw data when adjusted effect estimates were not available
  - ☒ AND they reported separate summary estimates for RCTs and NRSI separately when both were included in the review

- ☒ Yes
- ☐ No
- ☐ No meta-analysis conducted

12. If meta-analysis was performed, did the review authors assess the potential impact of RoB in individual studies on the results of the meta-analysis or other evidence synthesis?

For Yes:

- ☐ included only low risk of bias RCTs
- ☒ OR, if the pooled estimate was based on RCTs and/or NRSI at variable RoB, the authors performed analyses to investigate possible impact of RoB on summary estimates of effect.

- ☒ Yes
- ☐ No
- ☐ No meta-analysis conducted

13. Did the review authors account for RoB in individual studies when interpreting/ discussing the results of the review?

For Yes:

- ☐ included only low risk of bias RCTs
- ☒ OR, if RCTs with moderate or high RoB, or NRSI were included the review provided a discussion of the likely impact of RoB on the results

- ☒ Yes
- ☐ No

14. Did the review authors provide a satisfactory explanation for, and discussion of, any heterogeneity observed in the results of the review?

For Yes:

- ☐ There was no significant heterogeneity in the results
- ☒ OR if heterogeneity was present the authors performed an investigation of sources of any heterogeneity in the results and discussed the impact of this on the results of the review

- ☒ Yes
- ☐ No

15. If they performed quantitative synthesis did the review authors carry out an adequate investigation of publication bias (small study bias) and discuss its likely impact on the results of the review?

For Yes:

- ☒ performed graphical or statistical tests for publication bias and discussed the likelihood and magnitude of impact of publication bias

- ☒ Yes
- ☐ No
- ☐ No meta-analysis conducted

16. Did the review authors report any potential sources of conflict of interest, including any funding they received for conducting the review?

For Yes:	
☑ The authors reported no competing interests OR	☑ Yes
☐ The authors described their funding sources and how they managed potential conflicts of interest	☐ No

To cite this tool: Shea BJ, Reeves BC, Wells G, Thuku M, Hamel C, Moran J, Moher D, Tugwell P, Welch V, Kristjansson E, Henry DA. AMSTAR 2: a critical appraisal tool for systematic reviews that include randomised or non-randomised studies of healthcare interventions, or both. BMJ. 2017 Sep 21;358:j4008.
